# Extracorporeal Photopheresis Improves Graft Survival in a Full-Mismatch Rat Model of Kidney Transplantation

**DOI:** 10.3389/ti.2023.10840

**Published:** 2023-01-12

**Authors:** Gaston J. Piñeiro, Marta Lazo-Rodriguez, Pedro Ventura-Aguiar, Maria J. Ramirez-Bajo, Elisenda Banon-Maneus, Miquel Lozano, Joan Cid, Natalia Hierro-Garcia, David Cucchiari, Ignacio Revuelta, Enrique Montagud-Marrahi, Eduard Palou, Beatriu Bayés-Genís, Josep M. Campistol, Fritz Diekmann, Jordi Rovira

**Affiliations:** ^1^ Department of Nephrology and Kidney Transplantation, Hospital Clinic de Barcelona, Barcelona, Spain; ^2^ Laboratori Experimental de Nefrologia i Trasplantament (LENIT), Institut d’Investigacions Biomèdiques August Pi i Sunyer (IDIBAPS), Barcelona, Spain; ^3^ Red de Investigación Renal (REDINREN), Instituto de Salud Carlos III, Madrid, Spain; ^4^ Apheresis Unit, Department of Hemotherapy and Hemostasis, IDIBAPS, Hospital Clinic de Barcelona, Barcelona, Spain; ^5^ Department of Immunology, Hospital Clinic de Barcelona, Barcelona, Spain

**Keywords:** kidney transplantation, acute rejection, extracorporeal photopheresis, induction therapy, animal model

## Abstract

Extracorporeal photopheresis (ECP) is an immunomodulatory therapy based on the infusion of autologous cellular products exposed to ultraviolet light (UV) in the presence of a photosensitizer. The study evaluates the ECP efficacy as induction therapy in a full-mismatch kidney transplant rat model. Dark Agouti to Lewis (DA-L) kidney transplant model has been established. ECP product was obtained from Lewis rat recipients after DA kidney graft transplantation (Lew^DA^). Leukocytes of those Lew^DA^ rats were exposed to 8-methoxy psoralen, and illuminated with UV-A. The ECP doses assessed were 10 × 10^6^ and 100 × 10^6^ cells/time point. Lewis recipients received seven ECP infusions. DA-L model was characterized by the appearance of donor-specific antibodies (DSA) and kidney function deterioration from day three after kidney transplant. The dysfunction progressed rapidly until graft loss (6.1 ± 0.5 days). Tacrolimus at 0.25 mg/kg prolonged rat survival until 11.4 ± 0.7 days (*p* = 0.0004). In this context, the application of leukocytes from LewDA sensitized rats accelerated the rejection (8.7 ± 0.45, *p* = 0.0012), whereas ECP product at high dose extended kidney graft survival until 26.3 ± 7.3 days, reducing class I and II DSA in surviving rats. ECP treatment increases kidney graft survival in full-mismatch rat model of acute rejection and is a suitable immunomodulatory therapy to be explored in kidney transplantation.

## Graphical Abstract

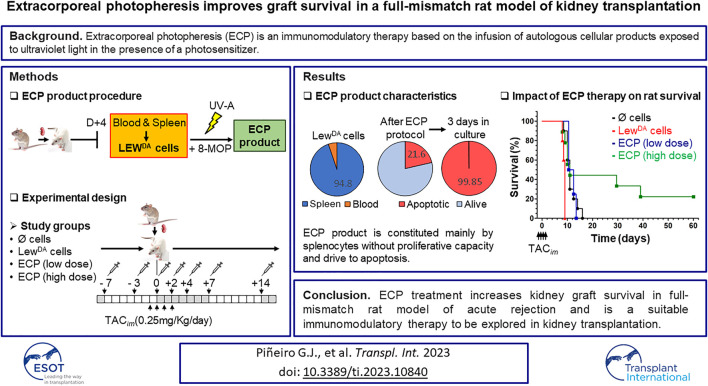

## Introduction

Kidney transplantation is the best therapeutic option for patients with end-stage renal (1–4), however, optimal control of the alloimmune response is still challenging. Antibody-mediated rejection (ABMR), and complications related to immunosuppression, are critical aspects that need to be addressed (5–7). To improve kidney graft survival, novel treatments should demonstrate a good security profile while attaining the desired control of the alloimmune response. In this sense, cell therapies, such as regulatory T cells (Tregs), regulatory macrophages (Mregs), Tolerogenic dendritic cells (TolDC), or Mesenchymal stromal cells (MSC), may be a suitable option providing control of the alloimmunity without increasing immunosuppression (8–10). Cell therapies are usually used as induction therapy to minimize the immune response against the graft as soon as possible or to minimize the immunosuppressive load reducing side effects.

Extracorporeal photopheresis (ECP) is an immunomodulatory therapy based on the infusion of autologous cellular products, obtained through leukopheresis, and exposed to ultraviolet light A (UV-A) in the presence of a photosensitizer, 8-methoxypsoralen (8-MOP). ECP has demonstrated to suppress various autoimmune and alloreactions without increasing overall immunosuppression and, therefore, without increasing infection rates (11–13).

The two main indications for ECP therapy are cutaneous T-cell lymphoma (CTCL) (14) and graft-versus-host disease (GvHD) (15, 16). Moreover, ECP has been previously applied in solid organ transplantation and demonstrated efficacy in (lung, heart and liver) improving response in steroid-resistant rejection episodes through no randomized clinical trials. In all of these transplants, it has also been used as an add-on therapy to standard immunosuppression to reduce the incidence of acute graft rejection during the first months following transplantation (17–26).

In kidney transplantation, most of the information is derived from case reports, small retrospective series, and a prospective study with short follow-up (18, 27–32). But the lack of evidence in kidney transplantation makes the use of ECP controversial.

The present study aims to evaluate the use of ECP as induction therapy in a full-mismatch kidney transplant model in rats. We hypothesized that ECP treatment could effectively prevent acute rejection, improving kidney allograft function and survival.

## Material and Methods

### Animal Model

Inbred male Dark Agouti rats (DA, RT1-A^av1^) were used as donors for allogenic renal transplantation for Lewis recipient rats (L, RT1-A^1^). The surgical technique was performed as previously described (33). Briefly, donor kidney procurement and kidney transplantation were performed under anesthesia with isoflurane. Donor kidneys were flushed with Celsior solution at 4°C and were stored in Celsior solution at 4°C until the implantation. Renal transplants were performed with an end-to-side anastomosis of the aortic stump of the donor kidney and recipient’s aorta, and between the recipient inferior vena cava and donor renal vein, respectively. Uretero-ureterostomy was performed with an end-to-end interrupted sutures technique. Recipient rats were bi-nephrectomized at transplantation.

The animals were kept at constant temperature, humidity, and at a 12-h light/dark cycle with free access to water and rat chow.

### Immunosuppressive Therapy

A short dose of tacrolimus (TAC) was established to favor initial graft function, avoiding a rapid loss due to acute rejection. TAC was administered during four consecutive days (−1, 0, +1 and +2 with respect to transplantation). To set the TAC dose to be used, the Lewis rat recipients were divided into three TAC dose groups, 0.1, 0.25, or 0.5 mg/kg.

### Detection of Donor-Specific Antibodies

DSA detection was performed in serum samples collected throughout the experiment. Dark Agouti donor rat splenocytes (5 × 10^5^ cells/sample) were suspended in MACS buffer for 10 min at room temperature and then incubated with 25 µL of recipient Lewis rat serum samples for 30 min. Cells were washed three times and then incubated with a panel of markers that include FITC-conjugated mouse anti-rat IgG (1:100 dilution), MHC class I (RT1A, OX-18 antibody) and MHC class II (RAT1B, HIS19 antibody) markers for a further 20 min. After washing (3 times), cells were suspended in 150 µL of MACS buffer and analyzed on FACS Canto II cytometer. As negative controls, cells were incubated with serum from non-immunized Lewis rats. The class I and II DSAs were quantify by mean fluorescence intensity (MFI) of FITC-IgG staining in cells that expressed MHC class I or II.

### Conventional Histology and Immunofluorescence

At 6 days of transplantation, a morphological and immunohistochemical study was performed in five Lewis rat recipients to identify the type of graft rejection. Formalin-fixed tissue was embedded in paraffin. Sections (3-μm thick) mounted on xylene glass slides (Dako, Carpinteria, CA) were used for immunohistochemistry. After antigen retrieval had been carried out, endogenous peroxidase blocking for 10 min in 3% hydrogen peroxide (Merck, Darmstadt, Germany) was performed before primary antibody incubation. The primary antibody, rat anti-C4d (Hycult Biotech, PA), was incubated overnight at 4°C. Envision system-specific anti-rabbit secondary antibody labeled with horseradish peroxidase polymer (Dako, Glostrup, Denmark) was applied for 1 h. All sections were counterstained with Mayer hematoxylin. Immunohistochemical procedure was performed at the same time to avoid possible day-to-day variations in staining performance. All images were acquired using an Olympus BX51 clinical microscope and DP70 digital camera and software (Olympus, Tokyo, Japan).

A renal pathologist evaluated hematoxylin/eosin, periodic acid Schiff and C4d stains to evaluate renal damage.

### Extracorporeal Photopheresis on Alloreactive Cells

**FIGURE 1:**
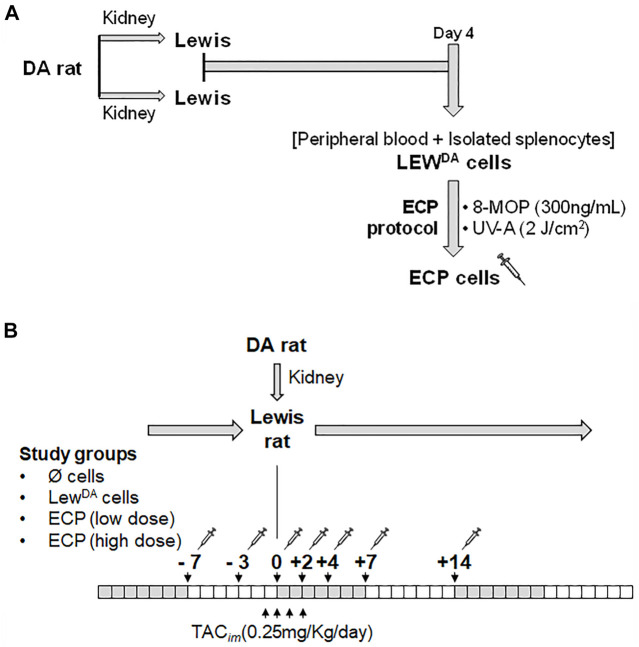
Generation of ECP cells and ECP induction therapy in kidney transplant rat model. **(A)** Generation of ECP cells. Each cell product batch contains LEW^DA^ leukocytes from 2 Lewis recipients’ rats. Four days after kidney transplantation, leukocyte cell suspention (LEW^DA^) were obtained from peripheral blood and spleen. LEW^DA^ cells were incubated with 8-MOP for 30 min, later cell suspension was illuminated with UV-A (2J/cm^2^) into MacoGenic (MACOPharma). **(B)** Extracorporeal photopheresis (ECP) treatment scheme in DA to Lewis kidney transplant rat model. Four groups were established according to the cell product used: without cells, Lew^DA^ cells, ECP at low dose (10^7^ cells/infusion) and ECP at high dose (10^8^ cells/infusion). DA, Dark Agouti rats; LEW, Lewis rats; Lew^DA^, leukocytes from sensitized Lewis rats; TAC_im_, intramuscular TAC injection; 8-MOP, psoralen; ECP, Extracorporeal photopheresis.

A total of 27 batches of Lewis rat leukocytes (Lew^DA^) were obtained from 54 transplanted rats (2 Lewis recipients per batch). Four days after DA-L kidney transplantation (Figure 1A), peripheral blood was collected and the spleen from 2 Lewis rat recipients were harvested, mashed, and passed through a cell strainer. Red blood cells were removed from both cell suspensions using RBC lysis buffer (Multispecies 10x, eBioscience), then were washed and counted. Both cell suspensions were characterized and subsequently pooled in a Lew^DA^ suspension.

The characterization of Lew^DA^ cells was performed by flow cytometry. Cell surface markers were stained with antibodies indicated in Supplementary Table S1, according to the instructions of the manufacturer. In all samples, Aqua Live/Dead fixable dead cell kit (Thermo Fisher Scientific, Waltham, MA, United States) was used unambiguously to remove dead cells. Flow cytometry analysis was performed on a FACS Canto II (BD Biosciences, Heidelberg, Germany). Data were analyzed using FlowJo software (Tree Star, Ashland, OR, United States). Overview of the gating strategy for T, NK and B cells has been shown in Supplementary Figure S1.

To perform the extracorporeal photopheresis, psoralen (8-MOP) at 300 ng/mL was incorporated to pooled Lew^DA^ cell suspension and 30 min later, cells were injected into UVA illumination bag XUV8501Q and then were illuminated with UV-A (2J/cm^2^) in MacoGenic G2 system (MacoPharma). The haematocrit was <1% in all ECP batches produced. At the end of illumination, ECP product was collected from the illumination bag and concentrated to infuse immediately into Lewis rats according to the planned dose. An aliquot of each ECP batch was analyzed to define cell viability and proliferation capacity.

### Analysis of the ECP Product

The viability of the ECP product and proliferative capacity in culture was analyzed using phytohemagglutinin (PHA-L) as a mitogen to trigger T-lymphocyte cell division. The viability of the ECP product was analyzed using Annexin V and viable dye stains. The proliferative capacity of ECP cells was determined by flow cytometry using carboxyfluorescein diacetate N-succinimidyl ester (CFSE) staining.

### Experimental Design

Lewis rat recipients were divided into four groups. All groups received intravenous TAC (0.25 mg/kg) for 4 days (−1, 0, +1 and +2 days with respect to transplantation). Group 1 (N = 10) received only TAC; group 2 (N = 5) in addition to TAC, received intravenous injections of 100 × 10^6^ Lew^DA^ cells (negative control, no photopherized cells), whereas groups 3 (N = 4) and 4 (N = 9), in addition to TAC, received intravenous injections of 10 × 10^6^ or 100 × 10^6^ ECP cells respectively. In groups 2 to 4, seven doses of cells (Lew^DA^ or ECP) were administered in phosphate-buffered saline at −7, −3, 0, +2, +4, +7 and +14-day respect to transplantation (Figure 1B). Serum blood urea nitrogen (BUN) and serum creatinine levels were measured to determine kidney function.

### Statistical Analysis

Statistical analysis was performed using the SPSS 21.0 statistics package. Univariate analysis using the log-rank test (Kaplan–Meier curves) was conducted to assess rat and graft survival (time from kidney transplantation to death). Values are given as mean ± SD. The Kruskal-Wallis or Mann-Whitney U tests were used where applicable.

### Ethics

This study was approved by and conducted according to the guidelines of the local animal ethics committee (Comitè Ètic d’Experimentació Animal (CEEA) from Universitat de Barcelona, Decret 164/1998).

## Results

We performed a full mismatch kidney transplantation rat model to determine ECP’s immunomodulatory properties as an addon to immunosuppression therapy *in vivo*. The study primary outcomes were graft and rat survival.

### Natural Course of the Allogeneic (DA-L) Kidney Transplantation Without Immunosuppression

**FIGURE 2:**
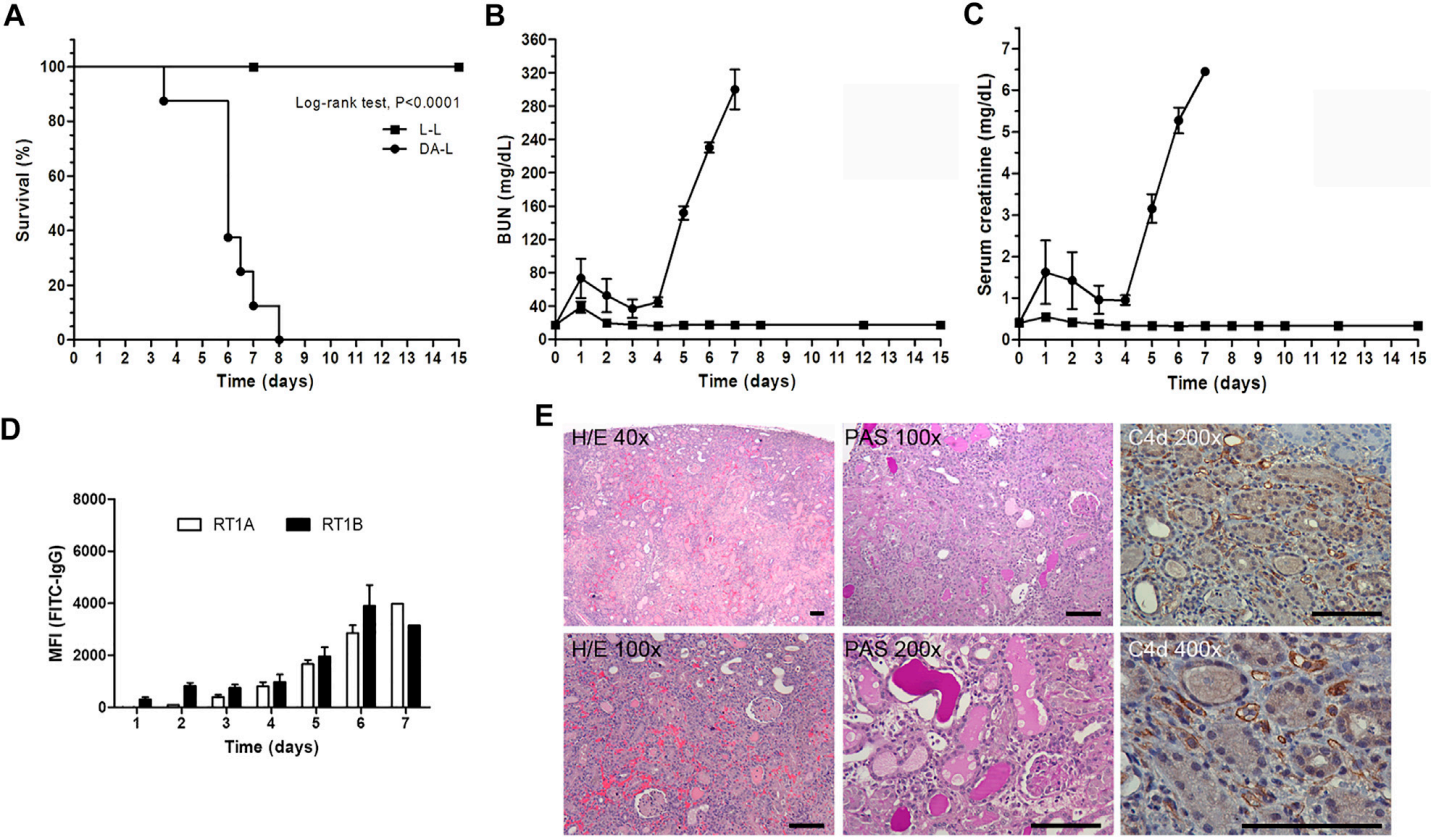
Characterization of DA-L rat kideny transplant model. **(A)** Rat survival analysis, isogenic (L-L, *n* = 11, square) versus allogenic (DA-L, *n* = 8, circle) kidney transplantation. **(B,C)** Renal function analysis including blood urea nitrogen (BUN) and serum creatinine, respectively. **(D)** Donor-specific antibodies analysis including rat MHC class I (RT1A) and MHC class II (RT1B). **(E)** Representatives’ images to illustrate kidney graft lesions observed in DA-L model at 6 days after transplantation. Hematoxylin/Eosin (H/E) and periodic acid Schiff (PAS) stains, and C4d immunohistochemistry were performed to determine that the rejection process observed in DA-LEW model is defined as an antibody-mediated rejection process. Scale bars are 100 μm in all pictures.

All Lewis rats that received a DA kidney graft died within 8 days after transplantation (mean survival 6.12 ± 1.28 days), whereas Lewis rats that received an isogeneic kidney graft (Lewis rat donor) survived until the last day of experiment (established at D+15 after transplantation). The characterization of the allogeneic (DA-L) kidney transplantation is shown in Figure 2.

The DA-L kidney transplant model was characterized by a slight deterioration of renal function on day +1 post-transplantation followed by an improvement in renal function and subsequently, from day +4, a rapid decline of renal function with an increase of BUN and serum creatinine levels, graft loss and rat death (Figures 2B,C).

Donor-specific antibodies (DSA) anti-RT1A and anti-RT1B were detected since day three with a progressive increase until day 7 (Figure 2D).

Kidney graft histology at 6 days after transplantation was consistent with acute antibody-mediated rejection (ABMR). Histology showed cortical necrosis, with peritubular capillaritis, and thrombotic microangiopathy. The interstitial infiltrates were mostly characterized by polymorphonuclear leukocytes and macrophages, and C4d deposition on peritubular capillaries was strongly positive at immunohistochemistry (Figure 2E).

### Impact of the TAC Therapy on the Allogeneic (DA-L) Kidney Transplantation

**FIGURE 3:**
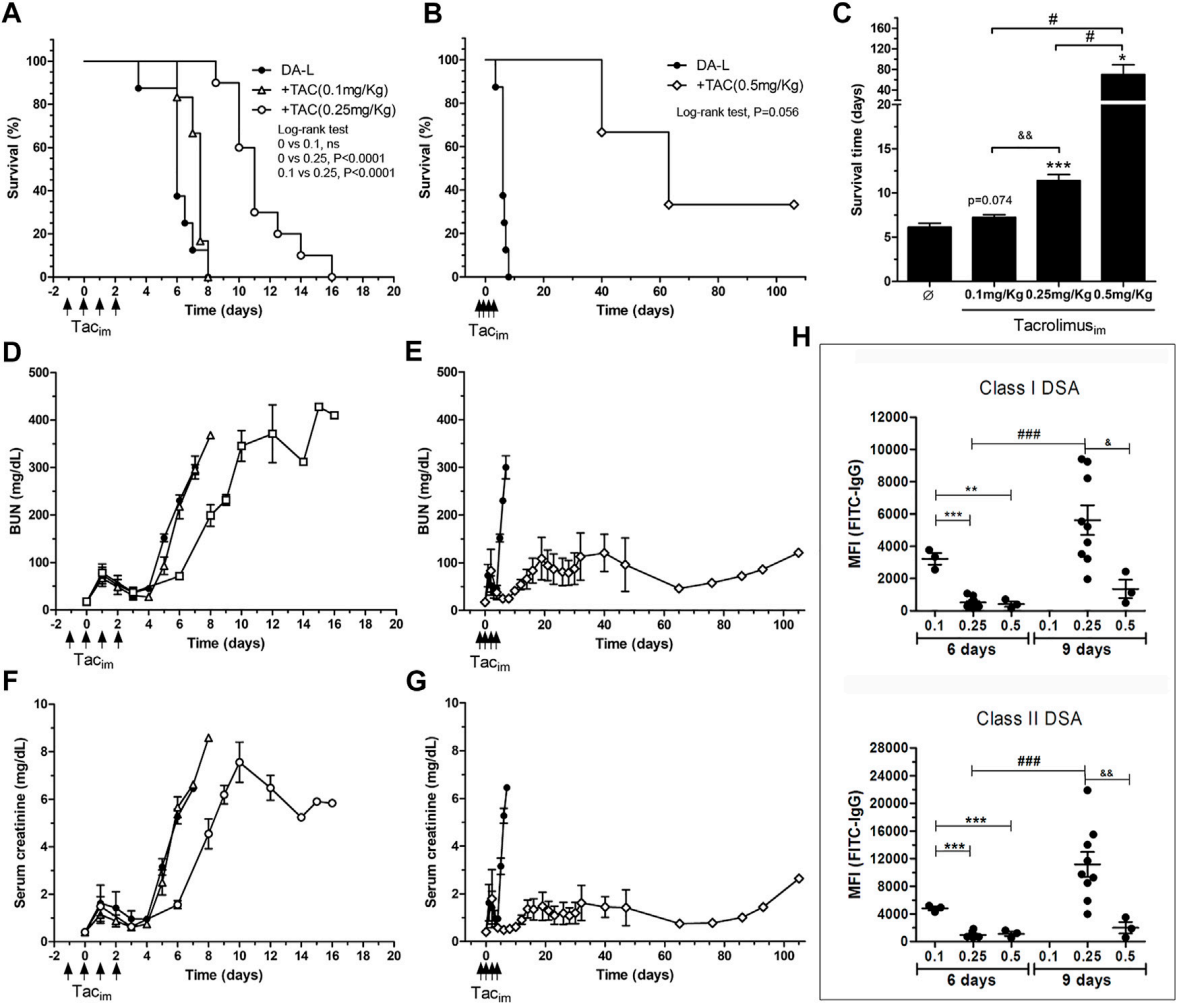
Immunosuppresion set-up in DA-L rat kideny transplant model. **(A,B)** Rat survival analysis, DA-L without immunosuppresion (*n* = 8, circle), with TAC at 0.1 mg/kg (*n* = 4, triangle), with TAC at 0.25 mg/kg (*n* = 10, square) and with TAC at 0.5 mg/kg (*n* = 3, diamond). **(C)** Mean survival time analysis. *Significantly different when compared to none treated group (*p < 0.05; **p < 0.01; ***p < 0.001). ^&^Significantly different when compared to TAC dose of 0.25 mg/kg. ^#^Significantly different when compared to TAC dose of 0.5 mg/kg. **(D–G)** Renal function analysis including blood urea nitrogen (BUN) and serum creatinine. **(H)** Quantification of class I and class II donor-specific antibodies (DSA) at 6 and 9 days after transplantation. *Significantly different when compared to TAC dose of 0.1 mg/kg (*p < 0.05; **p < 0.01; ***p < 0.001). TAC dose of 0.1 mg/kg. ^&^Significantly different when compared to TAC dose of 0.25 mg/kg. ^#^Significantly different when compared each group a different time point. TAC_im_, intramuscular Tacrolimus injection; MFI, mean fluorescence intensity.

The recipients with low dose of TAC (0.1 mg/kg) had a mean survival time of 7.25 ± .69, not different from the transplant model without immunosuppression (6.12 ± 1.28; *p* = 0.074) (Figures 3A,C). Recipients with medium and high doses of TAC (0.25 and 0.5 mg/kg) showed a significant increase of rat survival when compared to recipients without immunosuppression, 11.4 ± 2.21 (*p* = 0.0004) and 70.0 ± 32.8 (*p* = 0.0163), respectively (Figures 3A–C). BUN and serum creatinine levels, with a low TAC dose (0.1 mg/kg), were not different from the transplant model without immunosuppression. Whereas medium and high doses of TAC (0.25 and 0.5 mg/kg) preserved the renal function partially and showed a significant increase in survival (Figure 3).

The dose of 0.25 mg/kg of TAC was selected for the experimental design, and the low and high dose groups were discarded for the experimental model since they would make it difficult to show the effect of ECP treatment.

The effect of TAC doses on class I and II DSA levels is depicted in Figure 3H. On day 6 after transplantation, the dose of 0.1 mg/kg of TAC did not modify DSA levels compared with the model without immunosuppression (Figures 2D, 3H). The groups with the intermediate and high doses of TAC (0.25 and 0.5 mg/kg) showed levels of DSA significantly lower compared to the baseline model at day 7 post-transplantation. On day +9, class I and class II DSA MFI were significantly lower in the TAC 0.5 mg/kg group compared to TAC 0.25 mg/kg.

### Characterization of Lew^DA^ Cells

**FIGURE 4:**
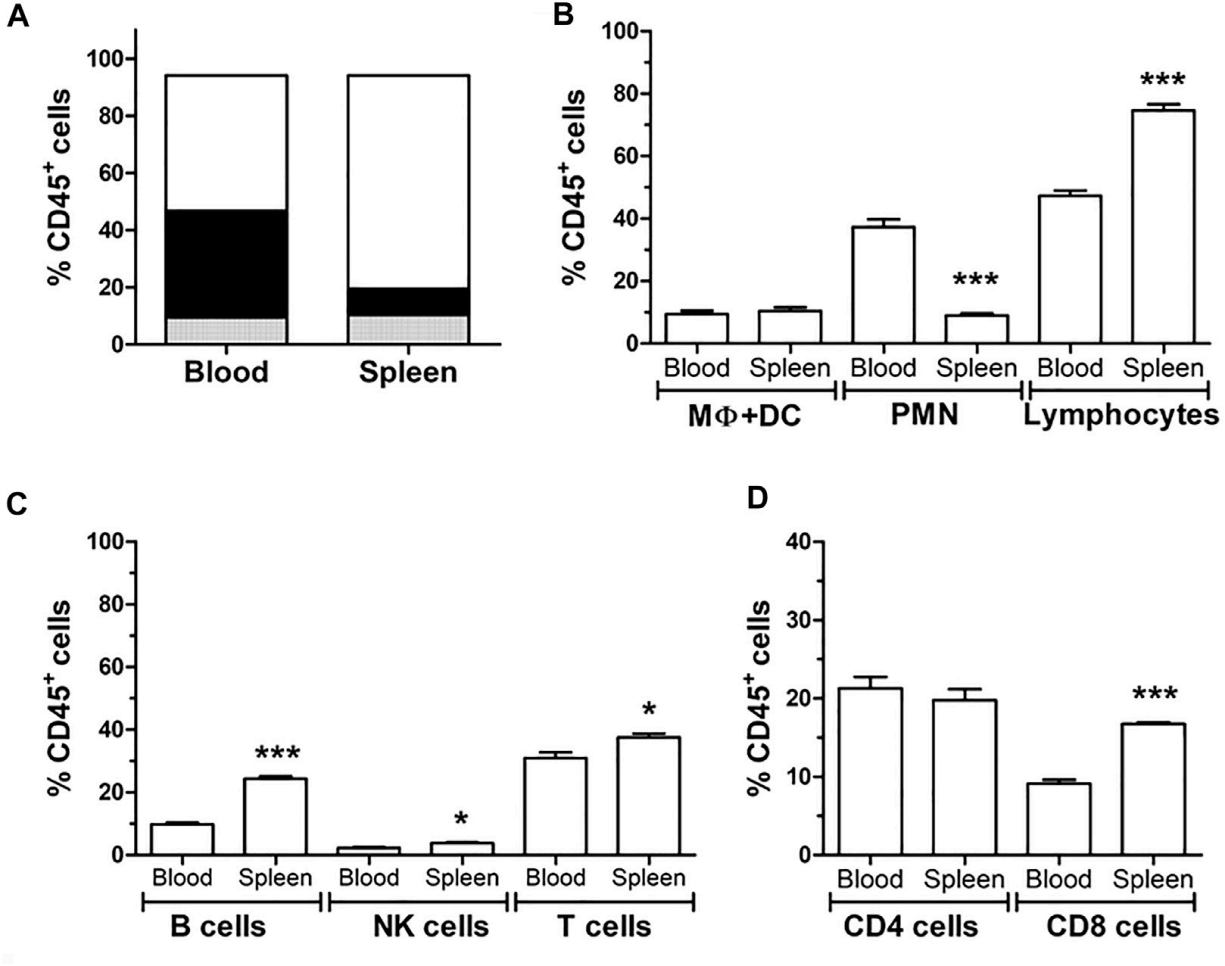
Characterization of spleen and blood circulating leukocytes by flow cytometry. **(A)** Distribution of the different leukocyte cell types; polymorphonuclear cells (PMN, black), monocytes (grey) and lymphocytes (white) according to Forward scatter (FSC) and side scatter (SSC). **(B)** Quantification of the different leukocyte types **(C)** Quantification of the different lymphocyte; B cells, NK cells and T cells due to CD45R (HIS24) and CD161 and CD3, respectively. **(D)** Quantification of CD3^+^ T cells in CD4^+^ and CD8^+^ cells. *Significantly different when compared to blood samples (*p < 0.05; **p < 0.01; ***p < 0.001).

Lew^DA^ cells from peripheral blood and spleen cell suspension were characterized by flow cytometry (Figure 4). T and B cells, NK cells, monocytes Lew^DA^ cells from both cell suspensions were pooled before the photopheresis procedure, concretely 5.24 ± 1.20% of Lew^DA^ cells were from peripheral blood in our 27 batches.

Lymphocytes predominate in the spleen cells and the polymorphonuclear cells in peripheral blood. In spleen cells, B lymphocytes and NK cells were more frequent than in peripheral blood. The concentration of CD4^+^ lymphocytes was similar between both origins, while CD8^+^ lymphocytes predominate in the spleen. The proportion of dendritic cells and macrophages was not different between peripheral blood and spleen.

### Characterization of ECP Cell Product

**FIGURE 5:**
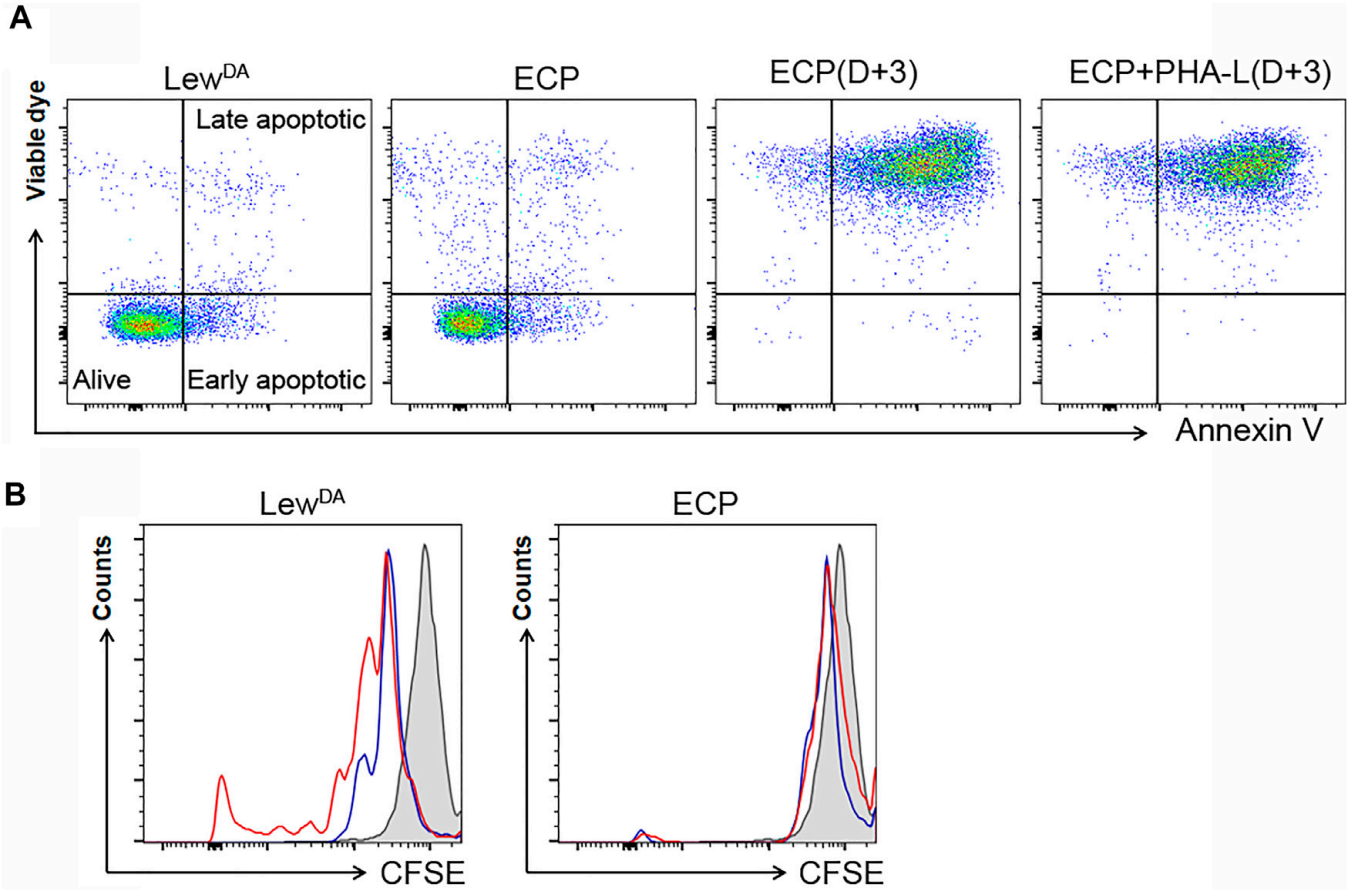
Characterization of the extracorporeal photopheresis product. **(A)** Impact of ECP on the viability of Lew^DA^. Representatives dot plots from Lew^DA^ cells, ECP cells, ECP cells cultured with full medium for 3 days and ECP cells cultured with phytohemagglutinin-L (PHA-L) for 3 days. In order to differentiate alive cells, early and late apoptotic cells, samples were stained with a viable die and Annexin V. **(B)** Impact of ECP on the proliferative capacity of Lew^DA^ splenonocytes and ECP product using CFSE staining. Left plot, Lew^DA^ splenocytes cultured for 3 days with or without PHA-L (red and blue lines, respectively). Right plot, ECP cells cultured for 3 days with or without PHA-L (red and blue lines, respectively). Gray shaded areas, cells stained with CFSE at Day 0.

The ECP cell product’s viability was assessed immediately after the photopheresis and after 3 days in culture with or without PHA-L as a mitogenic stimulus. ECP cell product seems to be alive (79.4 ± 1.4%) when the analysis was performed immediately after the photopheresis procedure compared to allogeneic Lew^DA^ cells (Figure 5A). However, when the ECP cell product was cultured for 3 days with or without PHA-L, about 95.2 ± 3.6% and 94.0 ± 0.2% of cells had to be considered apoptotic, respectively; moreover, just about 0.15 ± 0.06% and 0.14 ± 0.10% of cells remained alive, respectively (Figure 5A).

In order to evaluate the ECP cell product’s proliferative capacity, a mitogenic stimulus was added in the cell culture media. The photopheresis procedure completely avoided the proliferation observed in Lew^DA^ cells (Figure 5B).

### Impact of ECP on the Allogeneic (DA-L) Kidney Transplantation

**FIGURE 6:**
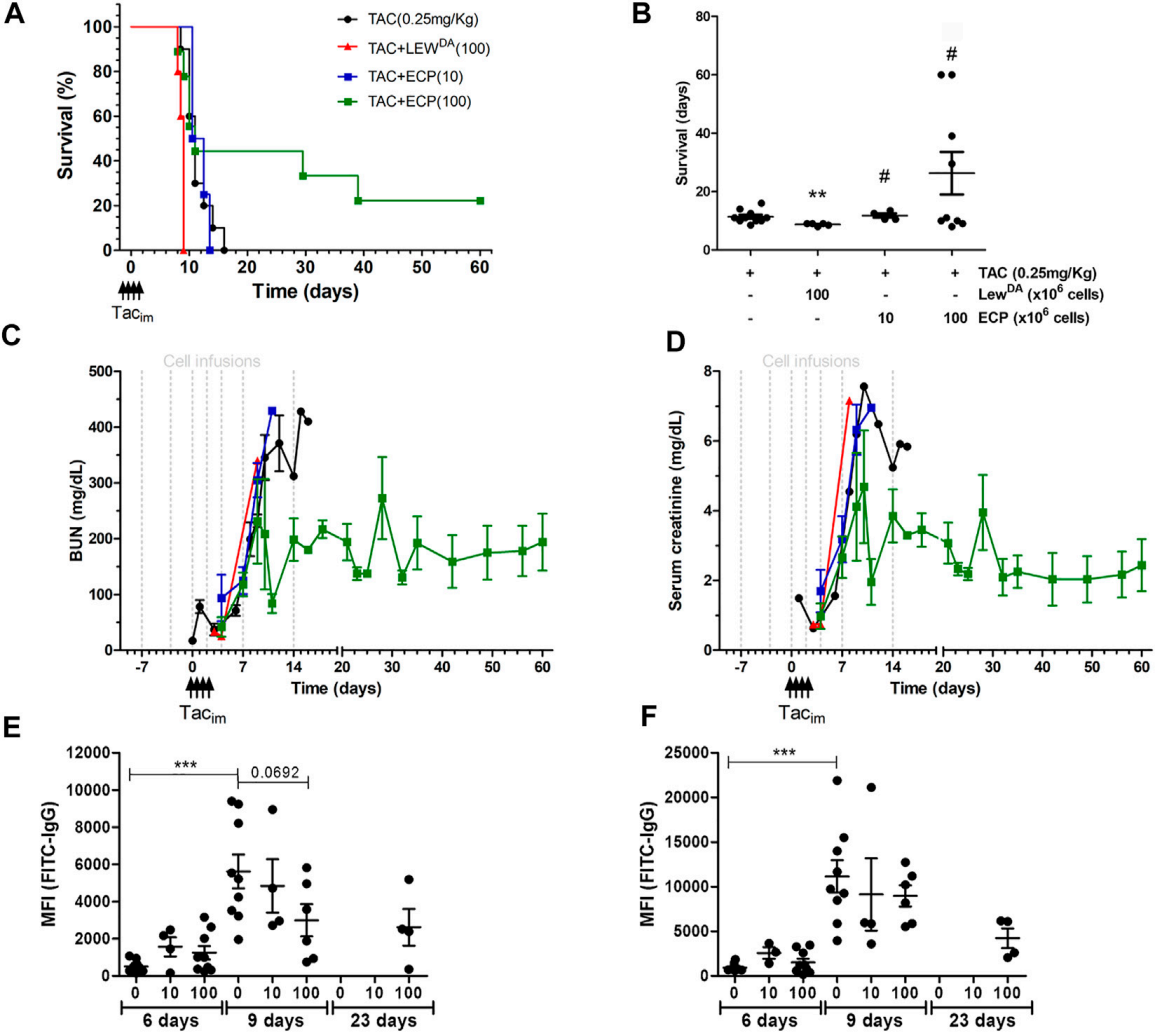
Impact of ECP product on DA-L rat kidney transplant model. **(A)** Rat survival analysis of the following groups of rats: DA-L+TAC (0.25 mg/kg) (*n* = 10, black cicle), plus Lew^DA^ (100 × 10^6^ cells, *n* = 5, red triangle), plus low dose of ECP (10 × 10^6^ cells, *n* = 4, blue square), and plus high dose of ECP (100 × 10^6^ cells, *n* = 9, green square) kidney transplantation. **(B)** Mean survival time analysis. *Statistical differences compare to TAC (0.25 mg/kg) group. **(C,D)** Renal function analysis including blood urea nitrogen (BUN) and serum creatinine. **(E,F)** Donor-specific antibodies analysis including rat MHC-class I (RT1A) and class II (RT1B). *Significantly different when compared to TAC dose of 0.25 mg/kg at 6 days. TAC_im_, intramuscular Tacrolimus injection.

The survival analysis is shown in Figures 6A,B. The infusion of Lew^DA^ cells from sensitized Lewis rats reduced the rat survival compared to TAC monotherapy group (8.7 ± 0.45 vs*.* 11.4 ± 2.21, *p* = 0.0012; Log-rank (Mantel-Cox) test, *p* = 0.0012). The illumination of Lew^DA^ cells, becoming ECP cells, blocks the acceleration of rejection observed in Lew^DA^ cells, all rats from the group TAC+ECP low dose (10 × 10^6^ cells) had equal mean survival time than TAC monotherapy group (11.75 ± 1.5 vs*.* 11.4 ± 2.21, P=NS; Log-rank (Mantel-Cox) test, P=NS) and increased mean survival time than TAC+Lew^DA^ (11.75 ± 1.5 vs*.* 8.7 ± 0.45, *p* = 0.0175; Log-rank (Mantel-Cox) test, *p* = 0.0074), whereas 44% of rats in the TAC+ECP high dose (100 × 10^6^) group survived until day 29 after transplantation. Mean survival time was prolonged in high doses of ECP until 26.28 ± 21.9 days being statistically different from TAC+Lew^DA^ group (*p* = 0.0308; Log-rank (Mantel-Cox) test, *p* = 0.0090).

Regarding renal function, BUN and creatinine were analyzed in Figures 6C,D, respectively. The infusion of Lew^DA^ cells accelerated the deterioration for kidney graft function compared to TAC monotherapy group. The high dose of ECP in combination with TAC stabilized the deterioration of kidney function in nearly 50% of rats (Figures 6C,D and Supplementary Figure S2), whereas a low dose of ECP did not confer any improvement in renal function compared to the TAC group.

The impact of ECP doses on class I and class II DSA is shown in Figures 6E,F. Both low and high ECP doses did not reduce the MFI of class I and class II DSA at day 6 after transplantation. The analysis at day 9 revealed that high doses of ECP in combination of TAC reduced partially class I DSA, concretely survivor rats had the lower class I and class II DSA levels. In the surviving rats at day 23 after transplantation, from TAC + ECP high dose group, the MFI for class I and II DSA were 2620 ± 989 and 4241 ± 1102, respectively.

## Discusion

In kidney transplantation, improving the management of antibody-mediated damage without increasing complications associated with immunosuppression is a crucial factor in graft and patient survival. At present, there are no FDA-approved treatments for acute or chronic antibody-mediated rejection, and plasma exchange with intravenous immunoglobulin constitutes the standard of care (34, 35). Even more, in chronic active ABMR, plasma exchange, and rituximab have been ineffective and are associated with a significant increase in severe infectious complications (36, 37). Rituximab has been also evaluated as induction therapy in kidney transplantation, however no convincing benefit was found and some safety concerns were identified (38, 39).

ECP is an attractive approach to prevent kidney graft rejection, given the absence of generalized immunosuppression or severe side effects.

DA-L kidney transplantation is a model characterized by early-onset antibody-mediated rejection with a rapid deterioration of renal function from day +4 after transplantation and short median survival of 6 days. To our knowledge, this is the first study to evaluate ECP in a full mismatch rat model of ABMR in kidney transplantation.

The efficacy of ECP on T-cell alloreactivity has long been proven, and in kidney transplantation, there are reports on the effectiveness of ECP in refractory T cell-mediated rejection (40). So, the efficacy of ECP on B- and plasma-cell mediated rejection mediatedantibody-mediated rejection is the remaining question. In the present study, *de novo* donor-specific antibodies (DSA) were detected from day three with a progressive increase, and kidney graft histology was consistent with ABMR. However, from an immune point of view, the development of high titers of DSA in 7 days, with the histological demonstration of antibody-mediated damage, fits better to a recall response. Although recipient rats have not been intentionally sensitized to donor antigens, we cannot rule out previous environmental exposure to cross-antigens with antigens from the donor rats.

In this model, a short course of four doses of TAC allows maintaining the graft’s initial function, increasing survival with a median of 11 days. The application of Lew^DA^ cells from sensitized rats accelerated the rejection process (8.7 ± 0.45 days), whereas the addition of high dose of ECP cells at (100 × 10^6^ cells, photopherized Lew^DA^ cells) prolonged mean survival time until 26.28 ± 21.9 days and stabilized the deterioration of kidney function in nearly 50% of rats. The addition of ECP to the treatment with a short course of TAC did not modify the expression of DSA in the initial period. However, on day 23 a decrease in DSA was evidenced compared to previous levels of MFI (day 9 after transplantation).

Previous evidence of ECP in solid organ transplantation, reported a significant decline in DSA and lung associated autoantibodies in pulmonary graft recipients with chronic allograft rejection in the form of the bronchiolitis obliterans (25). This decrease in antibodies occurred in conjunction with a reduction in pro-inflammatory cytokines and an increase in anti-inflammatory ones. A direct effect of ECP on DSA production cannot be ruled out, although indirect effects related to inflammatory factors appear to be involved. A decrease in B-cell activating factor (BAFF) levels and changes in B cell populations, has been identified as predictor of response to ECP treatment in GvHD (41, 42). Also, a mouse model of skin allograft showed that the infusion of splenocytes exposed to 8-MOP/UVA increased the number of IL-10^+^ regulatory B cells in circulation and promoted survival of the graft (43).

It has to be highlighted that a high dose of ECP was decisive in the stabilization of renal function, whereas a low dose of ECP did not confer any improvement in kidney function or rat survival compared to TAC monotherapy treatment. Our study suggests a dose-response or at least the possibility of a dose threshold; in fact, some individuals’ lack of response could be due to an insufficient dose.

Another debatable issue is the use of two pre-transplant doses. However, a point in favor of pre-transplant administration is the example of cellular therapies with mesenchymal cells (MSCs) where administration timing is of vital importance for their potency. Administration before the development of the inflammatory state increases the response to treatment (44).

This study has some limitations; immunosuppression was limited in time to highlight the effect of ECP in a small group of animals, making the model very aggressive. In humans, ECP therapy will be applied as add-on therapy to achieve immunomodulation. As usual in rat models, the cell source was mainly the spleen, while only 5.24% of ECP products were peripheral blood leukocytes. In contrast, ECP in humans is performed on peripheral blood leukocytes; the difference in the cell product can lead to different effects on the immune response that must be considered when the therapy moves to the clinic. In addition, the rate of antigen-specific cells in the ECP product should be known in future clinical trials, although we have not evaluated it in our study.

Another limitation of this study is the lack of evidence regarding ECP’s mechanism in the renal rejection model. However, this is the first description of a positive effect of ECP in a full mismatch preclinical model that could teach us about the clinical application and mechanisms of ECP as an induction therapy in kidney transplantation. The mechanism underlying ECP immunomodulations is poorly known and sometimes contradictory. ECP induces apoptosis on leukocytes, as we shown in our approach; when these apoptotic cells return to the patient they generate an immunomodulatory effect (45, 46). Briefly, apoptotic cells are cleared by macrophages and dendritic cells (DC), which then upregulate suppressor factors (e.g., TGF-β, IL-10, IDO, HO-1, HLA-G, and PGE2) and downregulate costimulatory molecules (CD80 and CD86), resulting in tolerogenic DCs (TolDC). TolDCs suppress the effector T cell activity and induce the production of regulatory T cells (iTregs) (47–49). Recently, Pilon et al. demonstrated that human apoptotic cells induced by ECP (Apo-cells) can inhibit allogeneic immune response that follows both direct and indirect alloantigen presentation (50).

Chronic antibody-mediated or cellular-mediated rejections are crucial in kidney transplant survival. However, it is necessary to develop new experimental models to address the utility of ECP in this particular problem.

In summary, ECP is an immunomodulatory therapy that appears to affect both the cellular and humoral arms of the immune response to the allograft. The established safety of ECP favours this approach as a potential treatment alternative in the setting of kidney transplantation. In our stringent model of acute kidney graft rejection, ECP was able to prolong kidney graft survival, preserving kidney function, although the effect of ECP on DSA production is not entirely clear. These promising results could pave the way for future clinical studies in kidney transplantation. In particular, our group is developing a clinical trial to elucidate the Impact of Photopheresis in the Prevention of Acute Rejection in Highly Sensitized *de Novo* Kidney Transplant Recipients (NCT04414735).

## Data Availability

The raw data supporting the conclusion of this article will be made available by the authors, without undue reservation.
