# Supplementary material for: Extracorporeal Photopheresis Improves Graft Survival in a Full-Mismatch Rat Model of Kidney Transplantation
Source: Transpl Int. 2023 Jan 12;36:10840. doi: 10.3389/ti.2023.10840 (PMC9876976; doi:10.3389/ti.2023.10840)
Supplement: Supplementary file 1 [file DataSheet1.PDF]

SUPPLEMENTARY MATERIAL

Extracorporeal photopheresis improves graft survival in a full-mismatch rat model of kidney transplantation

SUPPLEMENTARY TABLES

Table S1: Flow cytometry antibodies for lymphocyte characterization.

Marker	Dye	Cat. Nº	Clone	Company
**IgG**	FITC	11-4811	Polyclonal	eBioscience
**CD3**	FITC	11-0030	eBioG4.18	eBioscience
**CD45R (B220)**	FITC	11-0460	HIS24	eBioscience
**CD3**	PE	12-0030	eBioG4.18	eBioscience
**MHC-II**	PE	12-0920	HIS19	eBioscience
**CD45R (B220)**	PE	12-0460	HIS24	eBioscience
**CD3**	PerCP eFluor 710	46-0030	eBioG4.18	eBioscience
**CD161**	PerCP eFluor 710	46-1610	10/78	eBioscience
**CD90.1 (Thy1.1)**	PE-Cy7	25-0900	HIS51	eBioscience
**CD8**	PE-Cy7	25-0084	OX8	eBioscience
**CD3**	APC	12-0030	eBioG4.18	eBioscience
**MHC-I (RT1-A)**	APC	17-0921	OX18	eBioscience
**CD8**	APC	17-0084	OX8	eBioscience
**CD45**	APC-Cy7	47-0461	OX1	eBioscience
**CD4**	V450	561579	OX35	BD Horizon
**Aqua live-dead**	Pacific orange	L34957	---	Thermo Fisher

SUPPLEMENTARY FIGURES

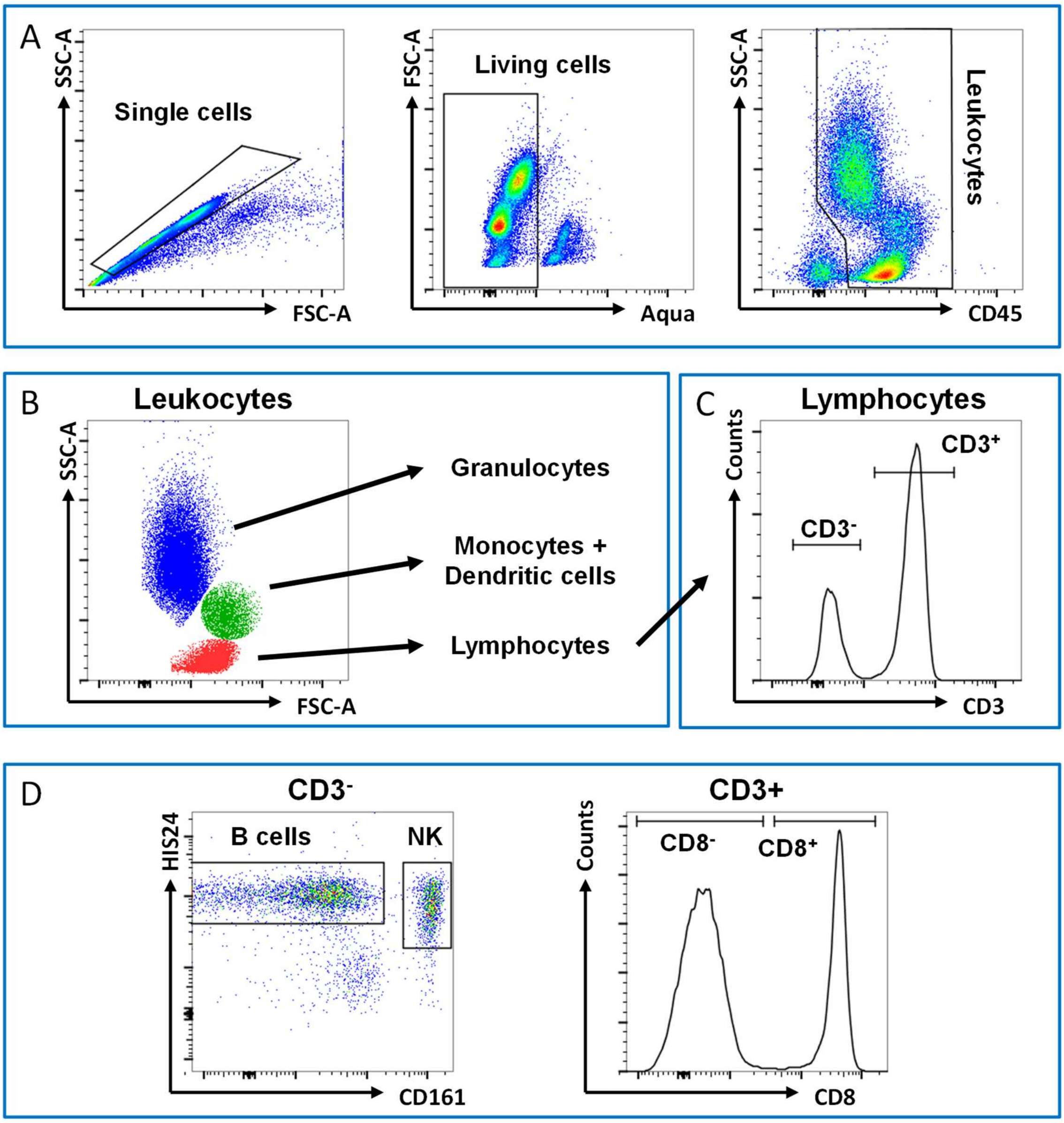

Figure S1. Overview of the gating strategy for the characterization of leukocytes from peripheral blood and spleen samples. (**A**) First of all, non-single events (forward scatter area versus forward scatter height) and death cells (LIVE/DEAD Fixable Dead Cell Stain Kits, ThermoFisher) were excluded; gating of leukocytes (forward scatter area versus CD45). (**B**) Leukocytes were differentiated in granulocytes, monocytes +dendritic cells and lymphocytes according FSC and SSC. (**C**) Lymphocytes were differentiated according CD3 staining; non T cells vs T cells. (D) Non T cells (or $CD3^-$ lymphocytes) were differentiated on B cells and NK cells due to CD45R and CD161 staing, whereas T cells (or $CD3^+$ lymphocytes) were differentiated in $CD8^-$ ($CD4^+$ T cells) and $CD8^+$ T cells.

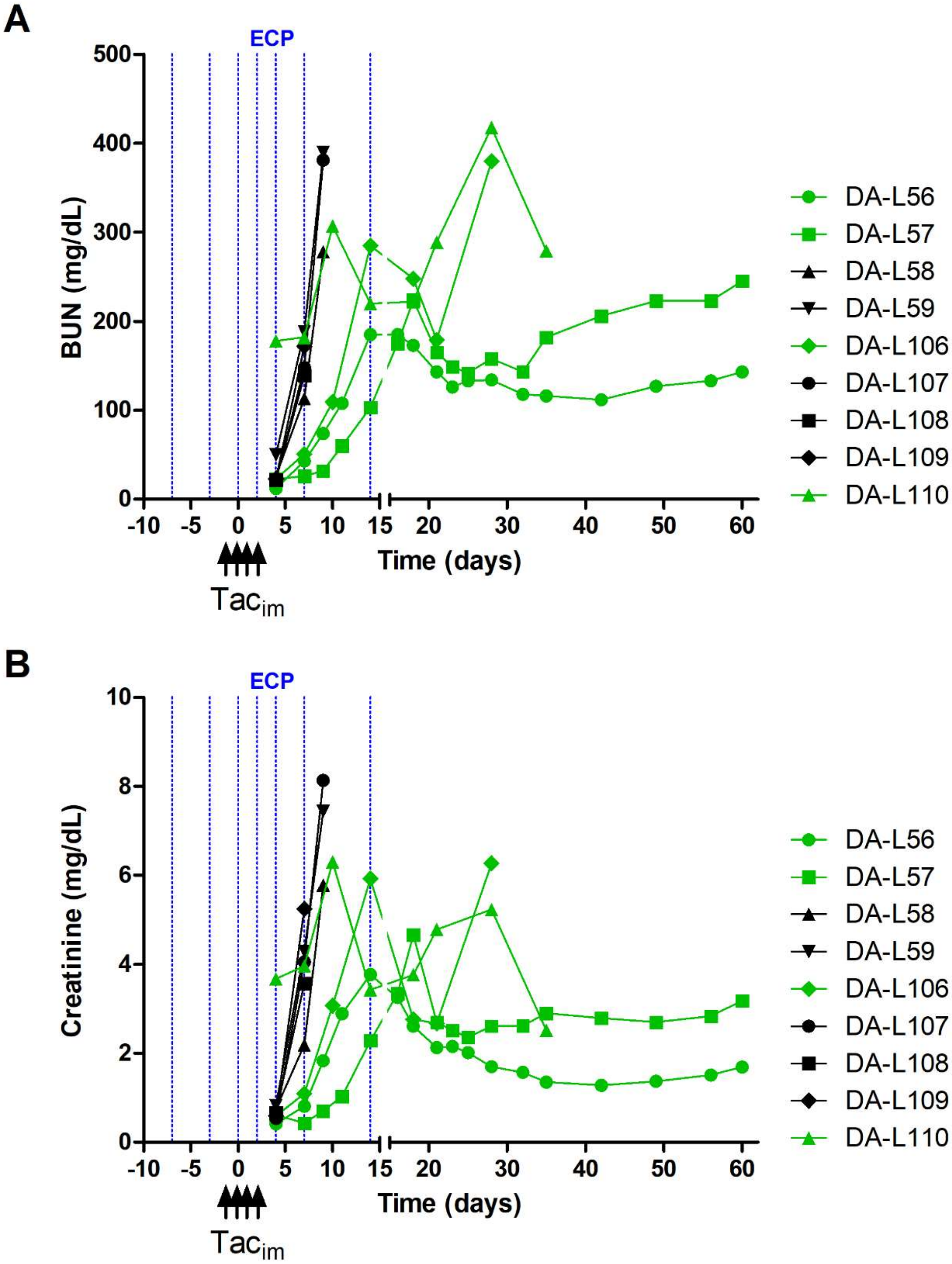

Figure S2: Impact of high dose of ECP (100x10^6 cell/infusion) on renal function for each rat. (A) Blood urea nitrogen (BUN) measurements. (B) Serum creatinine levels. Long-survivor animals are showed in green. Vertical blue lines indicate each ECP infusion, in these animals 100x10^6 cells/infusion. TAC_{im}, intramuscular TAC injection.
